# Amnesic Aphasia from Wound of Left Anterior Lobes of Cerebrum and Loss of Brain Substance

**Published:** 1882-09

**Authors:** E. Wyllys Andrews


### ARTICLE III.

### AMNESIC APHASIA FROM WOUND OF LEFT ANTERIOR LOBES OF CEREBRUM AND LOSS OF BRAIN SUBSTANCE. PARTIAL RECOVERY. E. Wyllys Andrews, A.M., M.D.

Michael Jordan, a young man æt. 25, while walking upon the railroad track near Joliet, Ill., was struck by a rapidly-moving train, and thrown from the track into a ditch, where he was found insensible. Dr. Chas. Richards, of Joliet, who was called to see the injured man, gives the following account of his condition:

"On May 16, 1881, I was summoned to Minonka to attend a man who had been struck by an engine. I found him in a comatose state, the result of a compound comminuted fracture of the frontal bone, upon the left side. Opening some three inches in length, and valvular. After carefully examining the wound, I found a portion of bone driven into the brain to the depth of one inch. At this examination about one tablespoonful of brain substance exuded, and was washed away while removing the clotted blood from the external parts. After removing the fragment of bone, I dressed the wound by bringing the integument together with sutures and applying cold water compress. I left him, expecting him to die before morning, but was notified on the following day that Michael Jordan was alive, and, to all appearances, better. I visited him about 2 p. m., and had him removed to Joliet, where he was under my care until he was removed to Chicago. At my second visit he seemed to know what was said to him, but could not speak. This condition lasted for ten days, when he was able to say some words, but could not converse. He soon became able to tell his name, and place of residence in Chicago, and could say "Yes," and some few other words. During the time he was under my treatment I removed, in all, about two ounces of brain matter, after which an abscess formed near the posterior edge of the fracture. This discharged freely, and was treated with carbolic acid and the continuous application of cold water. At this time Jordan's mind was, in a measure, lost, and he did not seem clearly to realize anything."

Jordan remained under the successful treatment of Dr. Richards until convalescence was well established, when he was brought to Chicago, and cared for in the County Hospital for a number of weeks. At the time of his discharge the wound was nearly healed, and, when he fell under my observation at the South Side Dispensary, in July, only a small granulating surface required any local treatment.

At this time the patient's cerebral symptoms were remarkable in showing the decided impairment of one function—viz.: articulate speech—without serious injury to any other faculty.

As experiments upon lower animals can furnish no knowledge of language as a localized function, clinical reports of such cases as this are the only sources of knowledge regarding the exact location of speech centers. The want of post mortem evidence in this case deprives it of much of its value in evidence ; but from external inspection, it is clear that the region of the excavation caused by the intruding fragment of bone, and the cavity of the subsequent abscess, must have been nearly in the usually-assigned region of speech, *i. e.*, the island of Reil, and the third frontal convolution on the left hemisphere of the cerebrum.

Jordan had been a man of weak intellect, bordering upon imbecility, so that his mental condition following the accident was but little worse than of old.

The cerebral symptoms began with coma, lasting for a few hours, followed by aphasia complete for ten days, and later slowly improving.

When first seen in Chicago, three months after the injury, the patient was quiet and passive in demeanor. Bodily vigor was unimpaired. The power of recollecting words was in a measure gone, so that utterance was extremely slow and labored, seemingly the result of intense effort and concentration of his feeble will. The patient disliked exceedingly to be put to the strain of uttering a response, and never spoke spontaneously. He seemed to labor intensely in uttering his monosyllabic replies. As to his mental power it was said by his family that he was not markedly worse than before the accident.

He presented upon the left side of the skull a depression back of the frontal eminence, two inches by an inch and a half, covering portions of the frontal and parietal bones. This corresponds to the size of the fragments of bone removed in the course of Dr. Richards' treatment. The scalp was depressed at this point one-half an inch, and pulsated visibly with the contents of the cranium.

The patient's friends informed me that at first he was subject to fits of anger, from which he is now mostly free, and that at such times the pulsations in the injured place were more noticeable, and the depression became a tumor.

As observed at a dozen different interviews, his aspect was that of childish imbecility. All his replies were rational and consequential, but his mind was seemingly a vacuum when he was not roused or called to concentrate his attention. There were no hallucinations. Morbid action seemed rather in the direction of weakened or depressed mental power. The only treatment adopted was occasional doses of bromides and cannabis, and a shield of gutta percha moulded upon a plaster cast to fit the concavity of the injured place and maintain a uniform pressure. This was designed partly to prevent the occurrence of outward bulging in the form of *hernia cerebri* or aneurism, and partly to guard the exposed brain from accidental puncture or concussion. The pressure of the apparatus, which was retained by an elastic cord worn under the hair, was a source of much comfort to the patient.

The loss of speech was now the most prominent symptom in which Jordan's condition differed from that before the injury. In January, 1882, a considerable improvement had taken place in the use of words, so that an intelligent answer would promptly be returned by the patient to any question requiring only "yes" or "no." Other questions, also, would be responded to with short replies, and there never appeared to be any incoherence in his expressions. A vocabulary of between one hundred and fifty or two hundred words seemed to be at his command, but any word would be repeated when given him without any defect of articulation. In February, since which time the patient has not been seen by me, the improvement was still more marked, the man's command of words being evidently more perfect as compared with his previous condition, and resembling that of a child in that he spoke mainly the names of objects without linking them into sentences.

Inasmuch as nearly total destruction of the left region of speech must have taken place, it is probably to be concluded that the right anterior cerebral lobes must have taken on the same function in a compensatory degree, unless it be supposed that in this case the injury of the island of Reil was something in the nature of plastic or other deposit, which became absorbed in the later stages of the recovery.

